# The application of weighted gene co-expression network analysis and support vector machine learning in the screening of Parkinson’s disease biomarkers and construction of diagnostic models

**DOI:** 10.3389/fnmol.2023.1274268

**Published:** 2023-10-16

**Authors:** Lijun Cai, Shuang Tang, Yin Liu, Yingwan Zhang, Qin Yang

**Affiliations:** ^1^Department of Pathophysiology, College of Basic Medical Sciences, Guizhou Medical University, Guiyang, Guizhou, China; ^2^Department of Neurology, The Affiliated Hospital of Guizhou Medical University, Guiyang, Guizhou, China

**Keywords:** Parkinson, weighted gene co-expression network analysis, support vector machine, immune microenvironment, biomarkers

## Abstract

**Background:**

This study aims to utilize Weighted Gene Co-expression Network Analysis (WGCNA) and Support Vector Machine (SVM) algorithm for screening biomarkers and constructing a diagnostic model for Parkinson’s disease.

**Methods:**

Firstly, we conducted WGCNA analysis on gene expression data from Parkinson’s disease patients and control group using three GEO datasets (GSE8397, GSE20163, and GSE20164) to identify gene modules associated with Parkinson’s disease. Then, key genes with significantly differential expression from these gene modules were selected as candidate biomarkers and validated using the GSE7621 dataset. Further functional analysis revealed the important roles of these genes in processes such as immune regulation, inflammatory response, and cell apoptosis. Based on these findings, we constructed a diagnostic model by using the expression data of FLT1, ATP6V0E1, ATP6V0E2, and H2BC12 as inputs and training and validating the model using SVM algorithm.

**Results:**

The prediction model demonstrated an AUC greater than 0.8 in the training, test, and validation sets, thereby validating its performance through SMOTE analysis. These findings provide strong support for early diagnosis of Parkinson’s disease and offer new opportunities for personalized treatment and disease management.

**Conclusion:**

In conclusion, the combination of WGCNA and SVM holds potential in biomarker screening and diagnostic model construction for Parkinson’s disease.

## Highlights

- This study was the first to utilize a combination of WGCNA and Support Vector Machine (SVM) to screen biomarkers and construct a diagnostic model for Parkinson's disease using multiple Parkinson's GEO datasets. Additionally, we have successfully developed a robust Parkinson's disease diagnostic model.
- This study reports for the first time the potential mechanisms and diagnostic value of FLT1, ATP6V0E1, ATP6V0E2, and H2BC12 in regulating the immune microenvironment in Parkinson's disease.
- This study demonstrates the significant value of constructing a diagnostic model using FLT1, ATP6V0E1, ATP6V0E2, and H2BC12, and their importance in the diagnosis of Parkinson's disease.

## 1. Introduction

Parkinson’s disease (PD) is a neurodegenerative disorder that primarily affects the elderly population, with a slightly higher incidence in males than females (Tansey et al., 2022). The exact cause is unknown but may involve genetic and environmental factors. Currently, there are no therapies that can reverse the progression of the disease (Lizama and Chu, 2021). Long-term pharmacological treatments face challenges such as declining efficacy and side effects, with the inability of neurons to regenerate posing a core obstacle to treatment (Liu et al., 2022). The pathogenesis of Parkinson’s remains incompletely understood, and reliable non-invasive biomarkers for early diagnosis are lacking (Surguchov, 2022). Emerging interventions like stem cell therapy and gene therapy are still under clinical investigation (Kline et al., 2021). In summary, Parkinson’s therapies are hampered by issues like drug dependence and difficulties with cell regeneration. Ongoing research and development of novel medications along with exploration of emerging treatment modalities is warranted to find effective therapies that can control disease progression.

Current research indicates close links between Parkinson’s disease and the immune system/inflammatory responses (Heidari et al., 2022). Neuroinflammatory reactions have been observed in brain tissues and peripheral blood samples of patients. Animal experiments also confirm microglial activation can exacerbate Parkinsonian symptoms (Haque et al., 2020; Zhang et al., 2021). Though anti-inflammatory treatments demonstrate some protective effects, the precise role of the immune system in Parkinson’s pathogenesis requires further investigation (Heavener and Bradshaw, 2022). Immunomodulatory therapeutic strategies for Parkinson’s are still in early exploratory phases. Overall, further elucidation of the interplay between Parkinson’s and the immune system may unveil novel therapeutic avenues for this disorder (Bjørklund et al., 2021).

Reliable biomarkers for early screening and diagnosis of Parkinson’s disease are still lacking at present. Various studies have attempted to identify specific biomarkers from peripheral blood, cerebrospinal fluid, imaging, genetic data analysis, etc., but with inconsistent results. Sensitivity and specificity of imaging techniques like PET need further improvement (Karayel et al., 2022). Screening methods utilizing olfactory testing and skin tissue samples are still in early phases. Using a combination of biomarkers may improve diagnostic performance but requires further optimization. Overall, non-invasive methods for early screening and diagnosis of Parkinson’s remain a central challenge and priority in current research. Obtaining reliable early diagnostic markers holds great significance for early detection and treatment of Parkinson’s disease (Atik et al., 2016; Angelopoulou et al., 2019). Therefore, the present study attempts to screen and validate potential PD biomarkers and molecular mechanisms through in-depth mining of multiple gene expression omnibus (GEO) datasets using weighted gene co-expression network analysis (WGCNA). The clinical diagnostic utility of identified biomarkers will also be evaluated using machine learning approaches.

## 2. Materials and methods

### 2.1. Data selection and preprocessing

**Figure 1:**
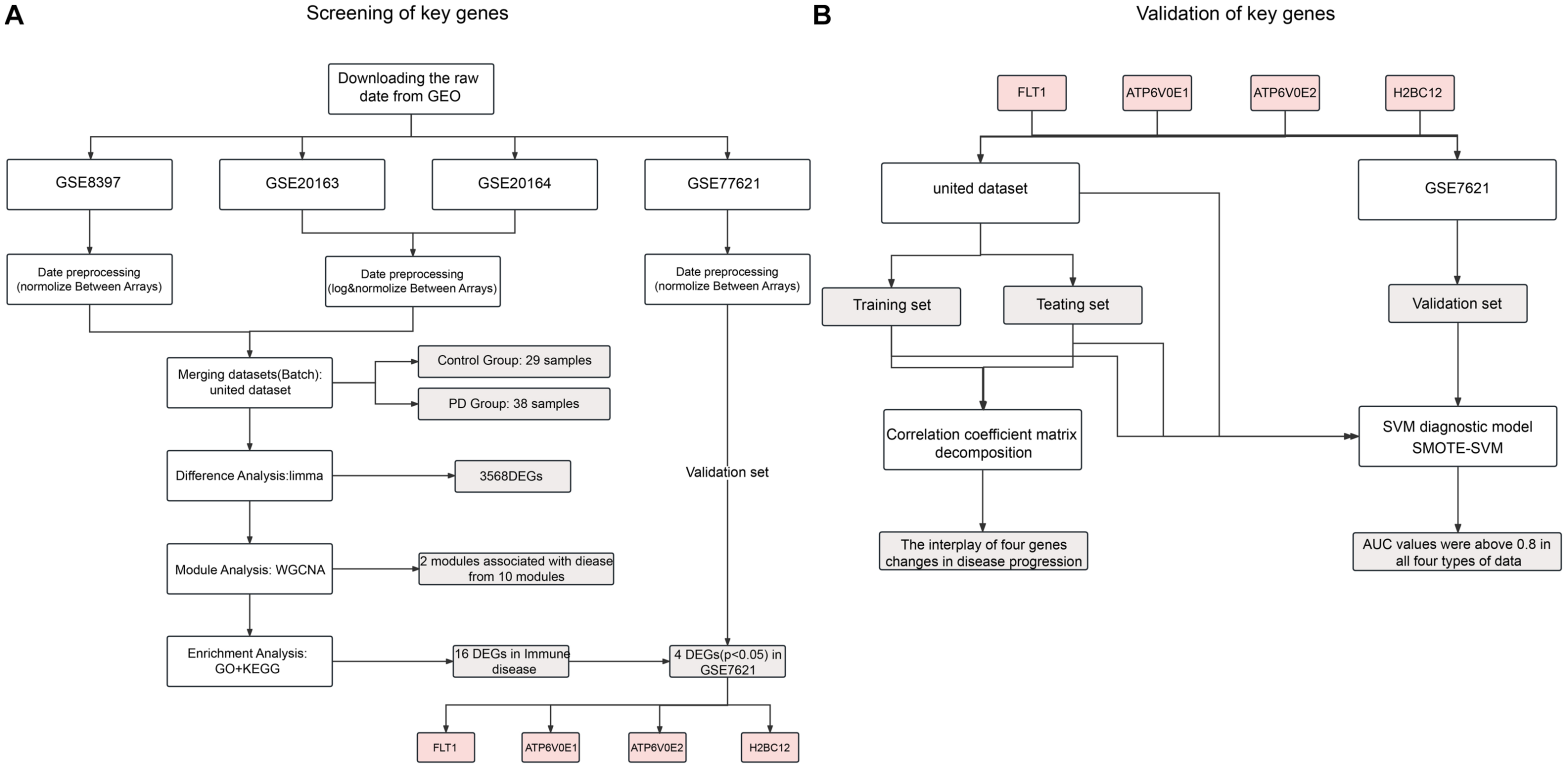
Flow chart of this study, respectively, for screening **(A)** and validation **(B)**.

In this paper, gene expression data for Parkinson’s disease were downloaded from the Gene Expression Omnibus.^1^ Altogether four datasets were screened out following the keywords “Parkinson’s disease” and “substantia nigra.” The microarray datasets GSE8397, GSE20163 and GSE20164 were obtained through the GPL96 platform. The raw expression data of the microarray data were selectively log2 transformed and normalized according to their numerical characteristics using the “affy” package in R software (version 3.48.3). Upon processing, the “sva” package (version 3.40.0) was used to remove batch effects from three datasets as the united dataset, including 38 PD patients and 29 controls. For the validation dataset, the GSE7621 obtained from GPL570 was preprocessed similarly, including 16 PD patients and 9 controls (Figures 1A,B).

### 2.2. Differential analysis to identify PD-related genes

Differential analysis of the united dataset was performed using the lmFit and eBayes methods from the “limma” package in R software (version 3.48.3). In order to identify a large number of differential genes with confidence, the *p* value < 0.05 was set as a threshold for the variation between the PD samples and the control samples. The captured genes were considered as the relevant genes for PD and further screened.

### 2.3. Construction of WGCNA for module analysis

The “WGCNA” package (version 1.71) was used to identify the expression patterns of all genes in the united dataset. First, all samples were clustered by the “hclust” function to check for the presence of outliers. If there were outliers, those would be removed and the remaining samples would be re-clustered to ensure the accuracy of the subsequent network construction. Then, the “pickSoftThreshold” function was used to calculate the soft threshold power. Next, a co-expression network for all genes was constructed and segmented using the “blockwiseModules” function. Finally, module-trait correlations were estimated using the correlation between the disease state of Parkinson’s disease as a clinical trait and module eigengenes. Two modules with significant positive and negative correlations with the clinical trait were selected, and the gene information corresponding to these modules was extracted for subsequent analysis.

### 2.4. GO and KEGG analysis of module genes

To explore the potential molecular function of the genes in the above selected modules, GO and KEGG analysis were performed. First, the GO analysis was performed by using the “clusterProfiler” package (version 4.0.5). Then, the KEGG pathways from David’s online analysis^2^ were visualized online using Bioinformatics,^3^ a tool that can perform secondary clustering of similar pathways. All the above results were significantly enriched with *p* value < 0.05.

### 2.5. Correlation coefficient matrix decomposition

To identify the hub genes with specific biological functions, a specific enriched pathway from KEGG analysis results was targeted. A set of genes with significant expression differences in the selected pathway was identified as the hub genes in GSE7621. After that, the Pearson correlation coefficients between the hub genes were calculated as $R_{con}$ and $R_{PD}$ for the control and PD samples in the united dataset. The “eigen” function in R software was used to decompose $R_{con}$ and $R_{PD}$ respectively to extract the corresponding eigenvalues λ and eigenvectors $\vec{X}$. They reflect the essence of the correlation coefficient matrix. Following the convention, the order of λ should be $\lambda_{1}\geq \lambda_{2}\geq \cdots \geq \lambda_{s}$ (s is the number of the hub genes). Conversion of all eigenvalues into percentages was performed using the formula $\lambda_{i}=\lambda_{i}/\overset{k}{\underset{1}{\sum \;\;\;}}\lambda_{k}$. Thus, the value of $\lambda_{i}$ is between 0 and 1, and the sum of them is 1.

### 2.6. Construction of the SVM diagnostic model

The package “sklearn” in Python (version 2.1) was used to build a support vector machine (SVM) diagnostic model for Parkinson’s disease. Aiming at this diagnostic model, the disease status of the sample can be determined more effectively based on the input of the hub genes. Here, we use the polynomial kernel function for the linear indivisibility feature of the united dataset. All samples from the united dataset were randomly divided into the training set (60%) and the testing set (40%), with the additional GSE7621 serving as an external validation set. For either data type input, the area under the ROC curve (AUC) was used to identify the accuracy of the model for disease classification. In addition, the ROC curve for the age characteristics of the samples in the united dataset were used as a control for our model.

### 2.7. SMOTE analysis

The SMOTE algorithm was used to oversampling the minority class samples. The main idea of the SMOTE algorithm is to randomly select a sample among the k nearest neighbors of each minority sample, and then interpolate between the lines of these two minority samples to generate a new minority sample. This process generates many new minority class samples, thus changing the imbalance ratio between majority and minority classes.

## 3. Results

### 3.1. The differential genes related to PD

**Figure 2:**
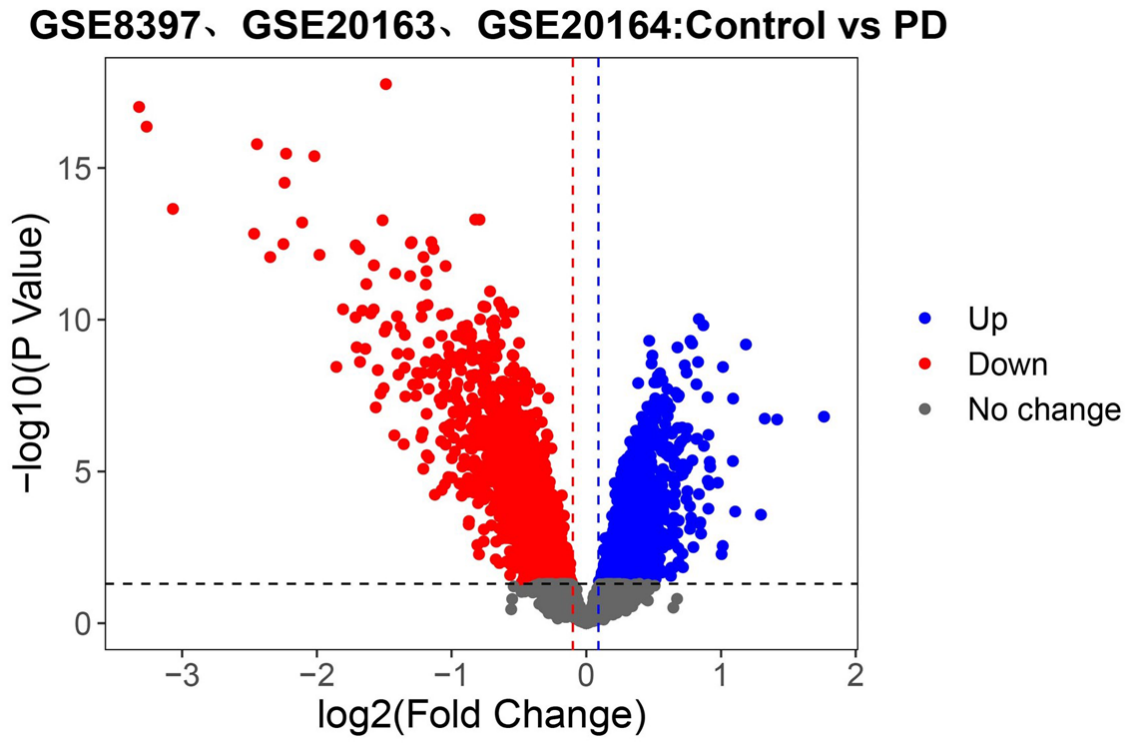
Identifying the differentially expressed genes (DEGs) related to PD in the united dataset. Volcano plot of all genes. Those with red dots represent up-regulated genes and those with blue dots represent down-regulated genes. The black dashed line, red dashed line and blue dashed line refer to the threshold of *p* value and logFC (Fold Change), respectively (Here, the threshold of logFC followed the setting of *p* value to obtain more differential genes). The gray dots delimited by the dashed lines represent genes that do not change.

To identify the set of genes with altered expression levels and high confidence in the alteration, the differential analysis was performed on the united dataset (GSE8397, GSE20163, and GSE20164; Figure 2). Samples from 38 PD patients and 29 normal nigrostriatal tissue differentially expressed a total of 3,568 genes, of which 1742 genes were up-regulated and 1826 genes were down-regulated in expression.

### 3.2. The modules most relevant to disease

**Figure 3:**
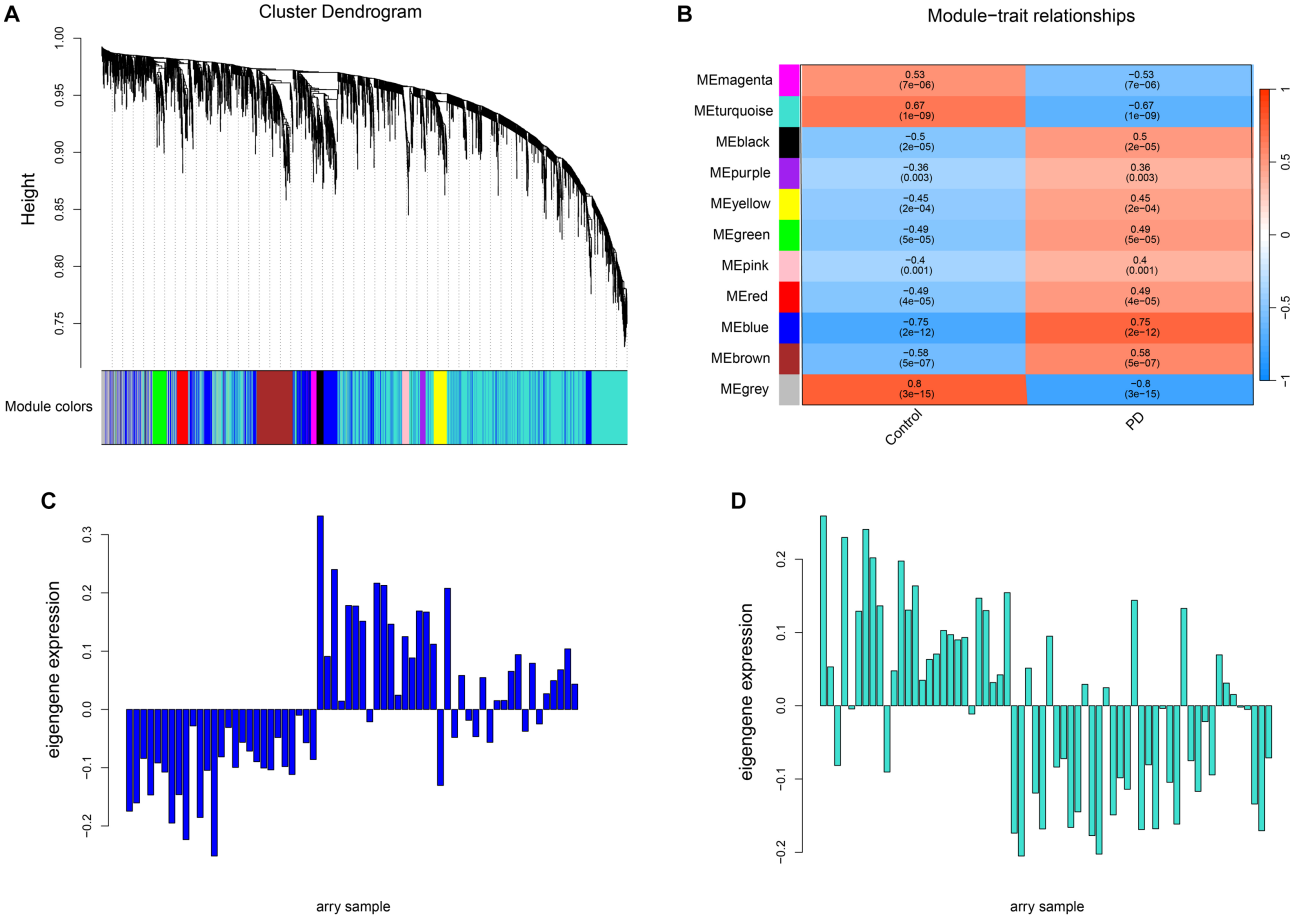
Screening the modules most related to PD through WGCNA. **(A)** The cluster dendrogram of 3,568 differential genes. A total of 10 co-expression modules were constructed with different colors at different degrees of similarity, where a module was represented by each color. **(B)** The correlation heatmap between the 10 modules and the Parkinson’s sample traits. The correlation coefficient and the corresponding confidence were shown in each unit. **(C,D)** The gene expression profiles in each sample.

Weighted co-expression network analysis (WGCNA) aggregates genes with similar expression patterns into one module and later the relationship with clinical traits can be explored through the module. Cluster all samples in the united dataset and remove the outlier samples GSM208642, GSM208630 and GSM208668 by setting the threshold value of Height to 50 (Supplementary Figure 1A). Afterwards, the compactness of the clustering between the remaining samples can be seen from the re-clustering plot (Supplementary Figure 1B), which is beneficial to improve the accuracy of module partitioning. Then β = 4 (scale-free R^2^ = 0.90) was chosen as the soft threshold for constructing the co-expression network (Supplementary Figure 1C). A total of 10 different modules appeared in the clustering tree (Figure 3A). Next, correlations were calculated based on the module eigenvalues with the clinical traits, that is, the status of the disease (Figure 3B). The blue module was significantly positively correlated with PD (r = 0.75; *p* = 5E-7) and the turquoise module was significantly negatively correlated with PD (r = −0.67; *p* = 1E-9). The expression of these two modular eigenvalues was plotted in each sample (Figures 3C,D), and a trend towards up-and down-regulation of expression levels was found overall, consistent with their correlation with the disease, respectively. Therefore, the blue and turquoise modules were identified as the most relevant to the disease and used for further analysis.

### 3.3. The differential genes in immune disease pathway for PD

**Figure 4:**
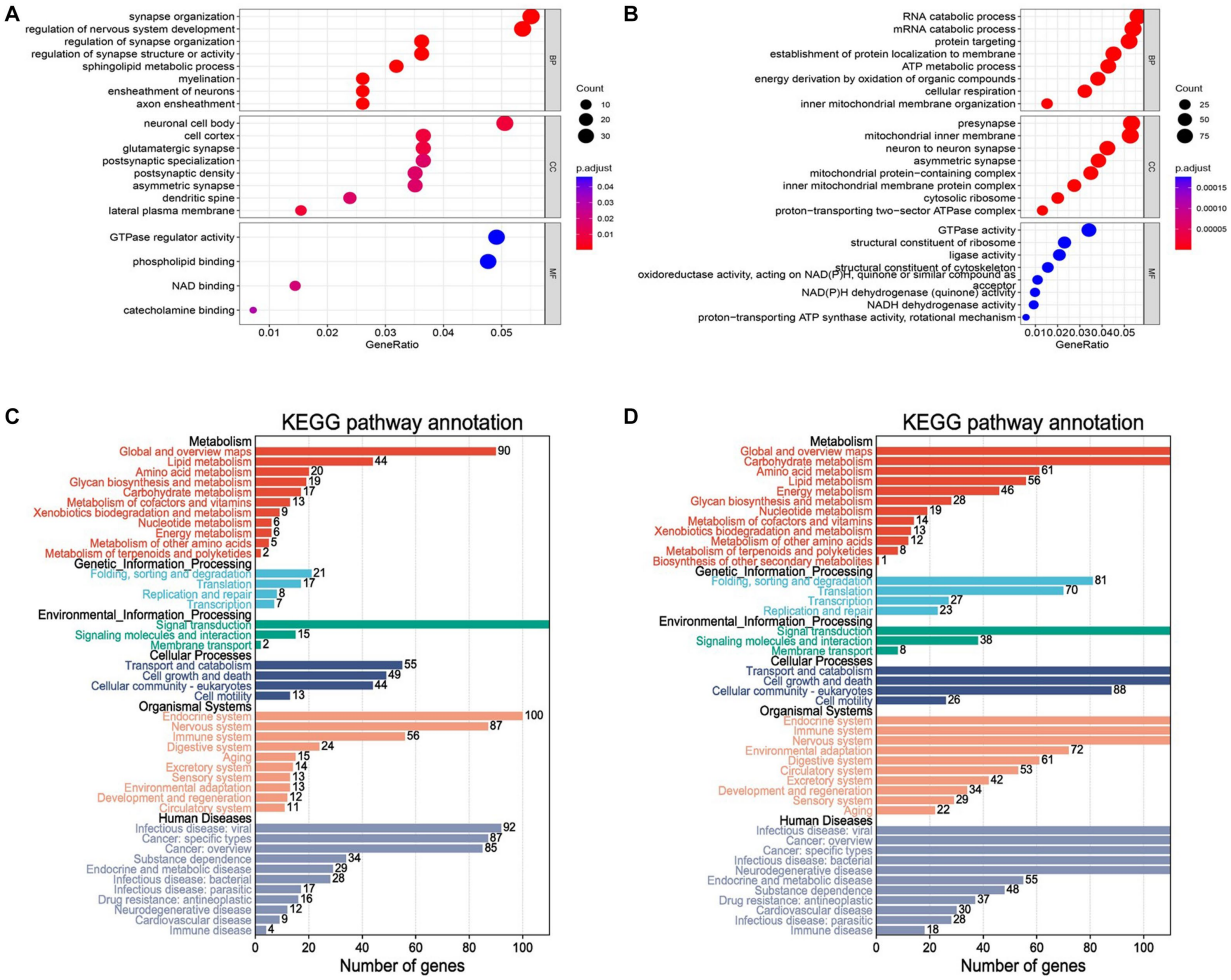
GO and KEGG analysis for blue and turquoise module genes. **(A)** The result of GO enrichment. The X-axis represents the GeneRatio (numbers of gene/gene size) enriched to the corresponding term. The larger the dot, the higher the numbers of gene enrichment to the term. The Y-axis indicates the name of the GO term. The color represents the adjusted *p* value. The redder the color, the smaller the adjusted *p* value. **(B)** The histogram of the blue and turquoise module genes by KEGG analysis. The results showed enrichment pathways of genes in metabolism, genetic information processing, environmental information processing, cellular processes, organismal systems and human diseases. **(C,D)** KEGG pathway enrichment analysis.

A set of 743 genes positively associated with Parkinson’s disease was obtained from the blue module above, denoted as $S_{blue}$. Also, there were 1805 genes negatively associated with Parkinson’s disease from the turquoise module, denoted as $S_{turquoise}$. The functional enrichment analysis was performed to further capture the pathogenic manner in which these genes were associated with the disease. GO enrichment results indicated that $S_{blue}$ were mainly involved in synapse organization, regulation of nervous system development and sphingolipid metabolic process in biological processes (BP) analysis (Figure 4A). Cellular component (CC) analysis revealed that $S_{blue}$ were primarily enriched in neuronal cell body, cell cortex and glutamatergic synapse. The top two enriched terms for $S_{blue}$ in molecular function (MF) were GTPase regulator activity and phospholipid binding. The enrichment results of $S_{turquoise}$ showed that the genes were mostly associated with RNA catabolic process, presynapse and GTPase activity in BP, CC and MF analysis (Figure 4B). In addition, KEGG analysis was performed for genes in $S_{blue}$ and $S_{turquoise}$ respectively, and the results of $S_{turquoise}$ were found to be more accurately enriched in neurodegenerative diseases (Supplementary Figures 2A,B). The re-clustering of KEGG analysis results by Bioinformatics was able to discover pathway affiliation as a whole (Figures 4C,D). The enriched genes of the immune disease pathway of $S_{turquoise}$ in human diseases were collected (Table 1). It is possible that these genes affect the disease onset and progression in PD through the biological process of immune response.

**Table 1:** The genes of the immune disease pathway of $S_{turquoise}$ in human diseases.

Class I	Class II	Pathway	Gene
Human diseases	Immune disease	Rheumatoid arthritis	ATP6V1F ATP6V0C ATP6V0E1 CXCL1 ATP6V0D1 ATP6V1B2 ATP6V1G2 ATP6V0B ATP6V1A ATP6V0E2 ANGPT1 ATP6V1H
Human diseases	Immune disease	Allograft rejection	HLA-G
Human diseases	Immune disease	Graft-versus-host disease	HLA-G
Human diseases	Immune disease	Autoimmune thyroid disease	HLA-G
Human diseases	Immune disease	Systemic lupus erythematosus	H2AC14 H2BC12

### 3.4. Alterations in the interplay between the hub genes

**Figure 5:**
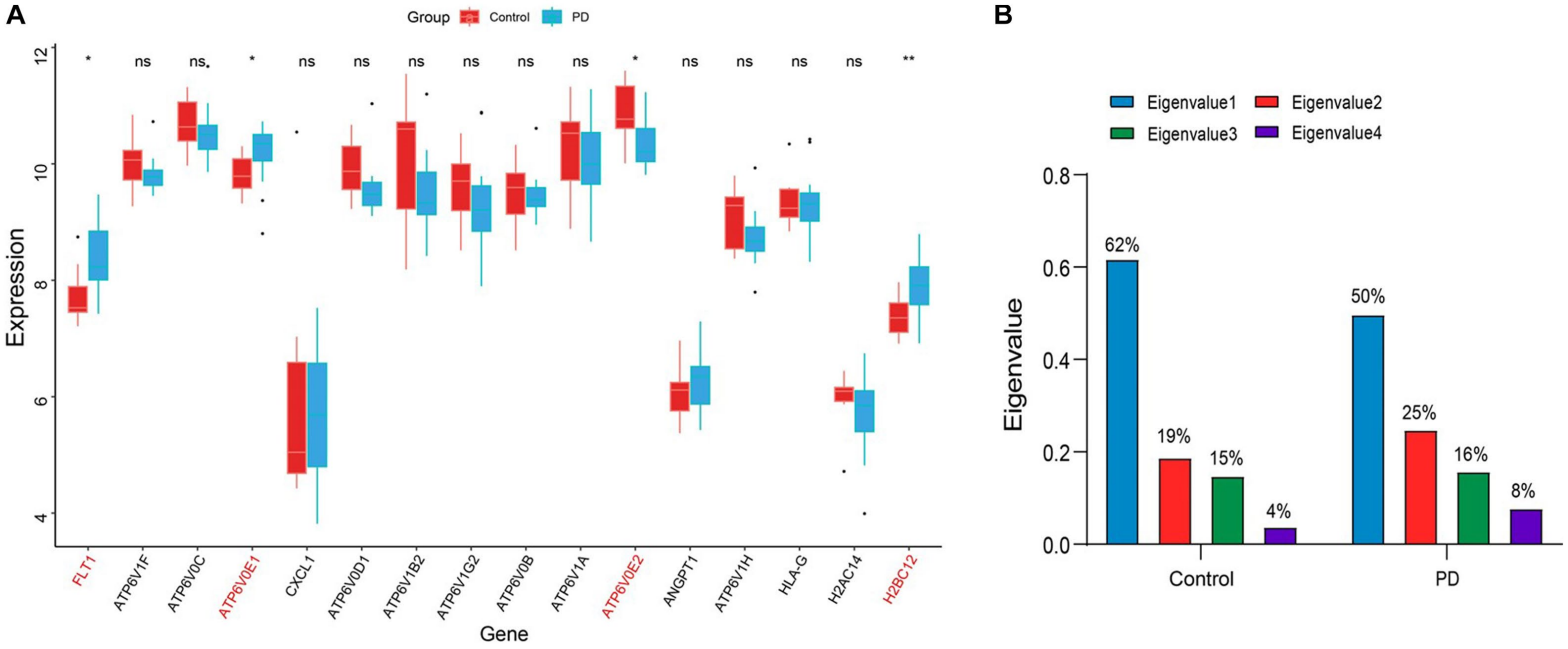
Identification of hub genes and their interactions. **(A)** The boxplot of the expression levels of PD-related genes in the immune disease pathway. The symbol * indicates significance *p* < 0.05 and conversely ns indicates no-significant change. The genes with significant change were marked in red font. **(B)** Histogram of the eigenvalue variations in the correlation coefficient matrix. The values on the bar chart have been converted to percentages, so the sum of eigenvalues is 1. The blue color indicates the largest eigenvalue, while the green color indicates the smallest eigenvalue.

To avoid the accidental screening of a single dataset, the validation dataset GSE7621 was used to identify the expression of 16 genes in Table 1. The results showed that only the alterations in gene expression levels of FLT1, ATP6V0E1, ATP6V0E2, and H2BC12 were credible (*p* value < 0.05; Figure 5A). Among them, the expression level of ATP6V0E2 was down-regulated compared to the controls, while the expression alterations of the remaining genes were up-regulated. These four genes were considered as the final hub genes, and the correlation coefficients between them were calculated (Supplementary Tables 1, 2). To explore the interplay variations between hub genes, eigenvalues and eigenvectors were obtained by matrix decomposition of the correlation coefficient matrix for control and PD stage. The results of the eigenvalues indicated a variation in the magnitude of the eigenvalue values in the PD stage compared to the control (Figure 5B). This suggested that the overall correlation between these four genes increased as the disease progressed, resulting in an elevated efficiency of the immune response.

In addition, the results of the eigenvectors showed the direction of alteration of the hub genes interaction (Tables 1, 2). For example, the eigenvector with the largest eigenvalue in the control group was [0.41, 0.59, −0.43, 0.54], contributing 62% of the matrix information. However, in the PD group, not only the eigenvalues changed, but also the symbols of the eigenvectors were reversed. This indicated that the expression pattern between the hub genes shifted from control group to PD group, leading to the deterioration of the disease. Among the elements, the first column of the eigenvector matrix corresponds to the largest eigenvalue, a most important element in the correlation coefficient matrix (Table 3).

**Table 2:** Eigenvectors of the correlation coefficient matrix in the control stage.

	V1	V2	V3	V4
1	0.41	0.82	−0.32	0.25
2	0.59	0.05	0.29	−0.75
3	−0.43	0.51	0.74	−0.02
4	0.54	−0.26	0.51	0.61

**Table 3:** Eigenvectors of the correlation coefficient matrix in the PD stage.

	V1	V2	V3	V4
1	−0.50	0.59	0.26	−0.59
2	−0.41	−0.74	−0.23	−0.49
3	0.52	−0.26	0.71	−0.39
4	−0.55	−0.22	0.62	0.52

### 3.5. Exploration into the association of hub genes with disease

**Figure 6:**
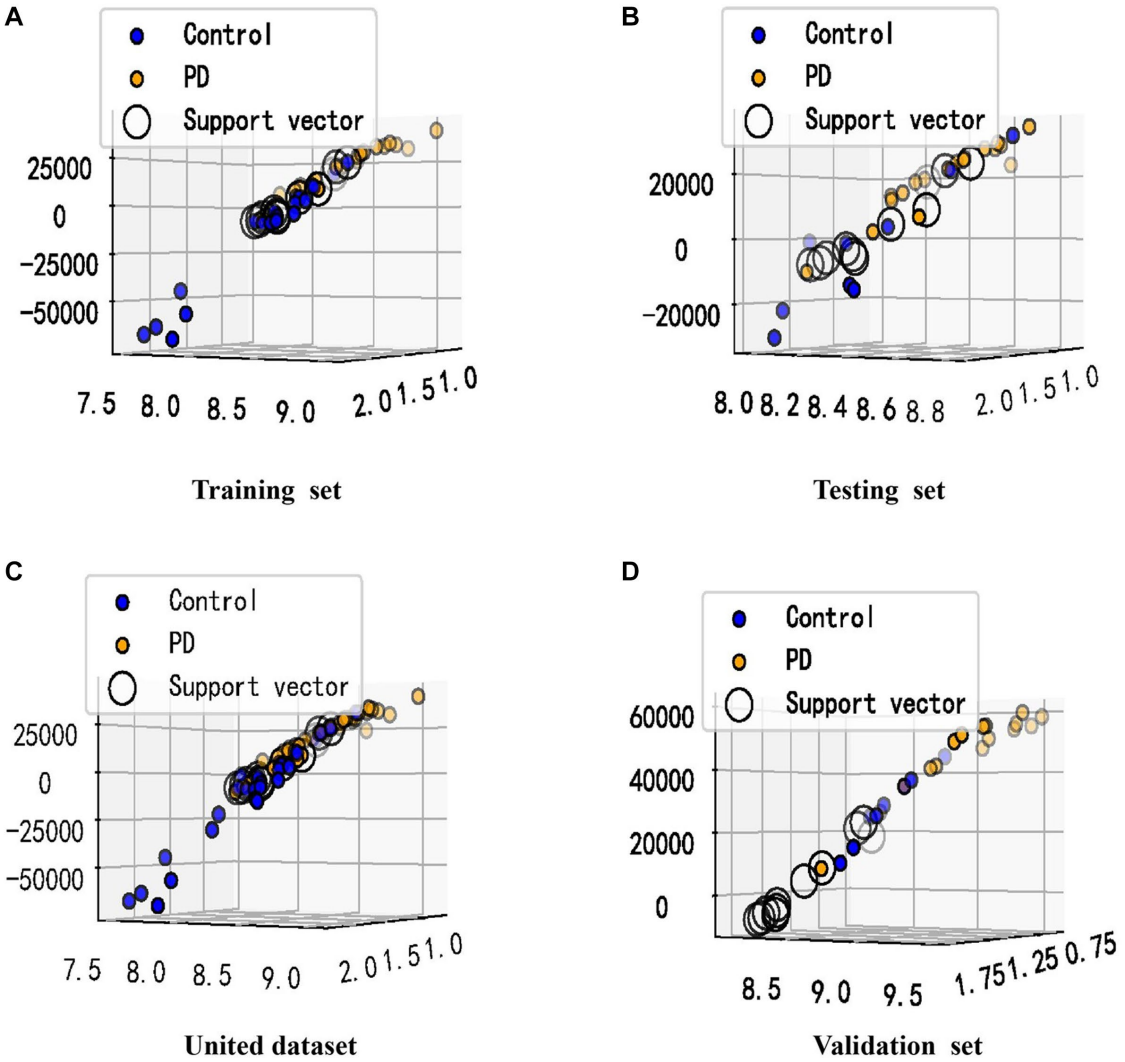
Visualization of the classification performance of the SVM model. **(A–D)** The samples from **(A–D)** were from the training set, testing set, united dataset and validation set, respectively. The X-axis represents the standard deviation of the hub genes, and the Y-axis represents the mean of the hub genes. The value of the Z-axis was obtained by Sklearn’s decision_function, which has been used to determine whether the sample belongs to the right or left side of the hyperplane, and the distance from the hyperplane. The support vector refers to the closest point to the hyperplane.

**Figure 7:**
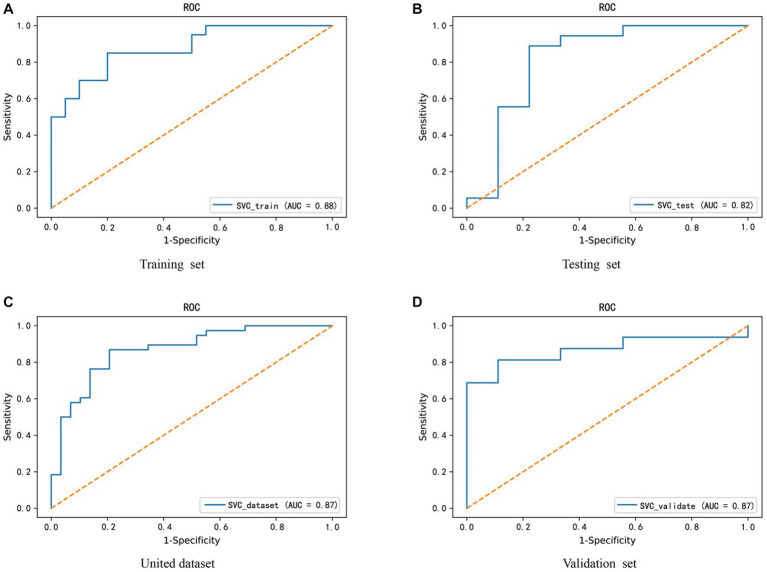
Credibility validation of the SVM model by ROC curves. **(A–D)** These are the model confidence for the training set, testing set, united dataset and validation set from **(A–D)**, respectively. The horizontal coordinate X-axis is 1 - specificity, also known as false positive rate. The closer the X-axis is to zero the higher the accuracy rate. The vertical coordinate Y-axis is called sensitivity, also known as true positive rate. The larger the Y-axis represents the better the accuracy rate.

Given the altered interactions between the hub genes, the association of such alterations with Parkinson’s disease was further explored. A training set from the united dataset was used to construct an SVM diagnostic model, using the mean and standard deviation of the hub genes as inputs to the model. The model was designed to find a hyperplane through the hub genes that could separate control samples from PD samples. The results revealed that the hyperplane of the model can accurately distinguish the samples in the training set, while it achieved a better division for the samples in the testing set, united set, and validation set (Figures 6A-D). In addition, to numerically know the credibility of the model, on the one hand, the ROC curves were used to validate the four types of datasets separately. The AUC values were all higher than 0.8, indicating the validity of the model to differentiate the control and patient groups (Figures 7A-D). On the other hand, the ROC curve plotted for the age characteristics of all samples (AUC = 0.74) indicated that the SVM diagnostic model was reliable (Supplementary Figure 3).

In addition, to avoid the experimental error caused by sample size, the SMOTE (Synthetic Minority Over-sampling Technique) algorithm was used to increase the proportion of minority class samples in the dataset, thereby reducing their impact on the classification effect of the SVM diagnostic model. Similarly, the AUC value of the ROC curve was used to evaluate the model’s classification effect (Supplementary Figure 4). We found that only the classification effect of the united dataset showed a slight change (from 0.87 to 0.86), thus the PD diagnostic model constructed by FLT1, ATP6V0E1, ATP6V0E2, and H2BC12 genes has certain predictive accuracy and practical guiding value in clinical decision-making.

## 4. Discussion

Parkinson’s disease is a neurodegenerative disorder primarily affecting middle-aged and elderly individuals. It has a global prevalence of approximately 4 million people, with over 1 million patients in China alone (Osborne et al., 2022). The main treatment modalities for Parkinson’s disease currently include pharmacotherapy and surgical interventions. However, their efficacy tends to decline over time, while the incidence of side effects increases (Levin et al., 2016; Reich and Savitt, 2019). Furthermore, recent research suggests a potential association between Parkinson’s disease and the immune system, including elevated levels of proteins in the blood and an increase in T-cell count (Nachman and Verstreken, 2022). Consequently, future investigations may explore the use of immunotherapy or alternative approaches for treating Parkinson’s disease. Early screening and diagnosis of Parkinson’s disease remain an active area of research. With advancements in technology, new biomarkers have been discovered that could aid in the early diagnosis of Parkinson’s disease. For example, studies have shown that specific proteins in cerebrospinal fluid can serve as diagnostic markers for Parkinson’s disease. Additionally, research is underway to explore other potential biomarkers, such as gene expression profiles. However, further validation and research are needed before these biomarkers can be effectively used in clinical diagnosis (Kluge et al., 2022). Genetic studies of PD have led us to realize that monogenic changes caused by single mutations of dominant or recessive genes play an important role in the analytical diagnosis of PD and are recommended for individual diagnosis (Selvaraj and Piramanayagam, 2019; Uslu et al., 2020). However, the screening and discovery of related susceptibility genes also restrict its development. Therefore, it is of great research value to find more molecular markers that may be related to PD by means of transcriptome screening, both in promoting the selection of its own application and in future mutation research. We had selected three GEO datasets for WGCNA analysis in order to identify relevant biomarkers and perform enrichment analysis of their functions and pathways. Furthermore, we have chosen another GEO dataset to validate and construct an SVM model using immune-related molecules to assess their potential diagnostic value in Parkinson’s disease.

The protein encoded by the FLT1 gene is a receptor for vascular endothelial growth factor, playing a crucial role in neurodevelopment and neuronal function. Several studies have suggested that mutations in the FLT1 gene may be associated with an increased risk of Parkinson’s disease (PD; Dharshini et al., 2021). Interestingly, our study identified a significant upregulation of FLT1 expression in PD patients, suggesting that the FLT1 gene could potentially serve as a therapeutic target for PD.

The ATP6V0E1 and ATP6V0E2 genes encode V0 subunits, which are key components involved in acid–base balance and lysosomal function. In the realm of immune microenvironment studies, these genes are thought to participate in processes such as regulation of immune cell acidification, lysosomal function, and antimicrobial activity. Several studies have indicated their significant roles in immune response and inflammation. However, the precise regulatory mechanisms and immune functions of these genes require further investigation for a comprehensive understanding (Fu et al., 2023; Zhu et al., 2023). Moreover, research has shown a potential genetic susceptibility of these two genes to Parkinson’s disease, but their functional implications necessitate further exploration and validation to elucidate their precise involvement in the pathogenesis of PD (Jin et al., 2012; Higashida et al., 2017). The H2BC12 gene encodes a subunit of histone H2B and has received relatively little attention in Parkinson’s disease research. Preliminary studies suggest that the H2BC12 gene may be associated with disease occurrence and progression; however, a more detailed mechanistic understanding needs to be established through further investigations (Jia et al., 2022; Zhou et al., 2022). Additionally, limited research has been conducted on its role within the immune microenvironment. Our research, for the first time, reveals its potential impact on PD progression through its regulatory effects on the immune microenvironment. Therefore, studies on the FLT1, ATP6V0E1, ATP6V0E2, and H2BC12 genes within the immune microenvironment are still in their early stages. Current research primarily focuses on exploring their associations with immune regulation, inflammatory responses, and immune cell functionalities. However, a thorough comprehension of their specific mechanisms of action and functions necessitates further investigation. At present, scholars have used TIMER and other tools to analyze the infiltration of immune cells in the field of oncology research, but we have not seen similar reports in PD research, but this will serve as a better guide for our future research (Liu et al., 2021; Wu et al., 2022; Zhang et al., 2022). We hope that future research endeavors will contribute to a more profound understanding of these genes’ roles within the immune microenvironment and their potential clinical applications.

In this study, we further utilized a training set from a combined dataset to construct an SVM diagnostic model using the mean and standard deviation of core genes as input features. The aim of this model was to find a hyperplane through the core genes to separate control samples from PD samples. The results showed that the hyperplane of the model accurately distinguished samples in the training set and achieved improved classification for the test set, combined set, and validation set samples. Additionally, to assess the reliability of the model quantitatively, we validated the four datasets using ROC curves and obtained AUC values greater than 0.8, indicating the effectiveness of the model in discriminating between control and patient groups. However, despite employing multiple analytical approaches to evaluate the diagnostic value of the model, future validation in large clinical cohorts is still necessary. In addition, with the increasing attention to epigenetics, whether these molecules are regulated by epigenetic mechanisms such as non-coding RNA or DNA methylation in the mechanism of disease still needs to be further explored. This remains one of our main directions for future research.

In conclusion, this study employed bioinformatics techniques to construct a diagnostic model for Parkinson’s disease. Our research provides important insights for the selection of early screening biomarkers and target identification for targeted therapy in Parkinson’s disease.

## Data availability statement

The original contributions presented in the study are included in the article/Supplementary material, further inquiries can be directed to the corresponding author.

## Ethics statement

The studies involving humans were approved by Ethics Committee of Guizhou Medical University. The studies were conducted in accordance with the local legislation and institutional requirements. Written informed consent for participation in this study was provided by the participants’ legal guardians/next of kin.

## Author contributions

LC: Conceptualization, Formal analysis, Methodology, Writing – original draft. ST: Conceptualization, Formal analysis, Investigation, Writing – original draft. YL: Data curation, Formal analysis, Software, Writing – review & editing. YZ: Data curation, Formal analysis, Software, Writing – review & editing. QY: Funding acquisition, Investigation, Supervision, Writing – review & editing.

## Footnotes

^1^ https://www.ncbi.nlm.nih.gov/geo/

^2^ https://david.ncifcrf.gov/

^3^ https://www.bioinformatics.com.cn/

## Abbreviations

WGCNA: Weighted gene co-expression network analysis
SVM: Support vector machine
PD: Parkinson’s disease
GEO: Gene expression omnibus
SVM: Support vector machine
CC: Cellular component
MF: Molecular function
