# Supplementary figures and images for: The application of weighted gene co-expression network analysis and support vector machine learning in the screening of Parkinson’s disease biomarkers and construction of diagnostic models
Source: Front Mol Neurosci. 2023 Oct 16;16:1274268. doi: 10.3389/fnmol.2023.1274268 (PMC10614158; doi:10.3389/fnmol.2023.1274268)

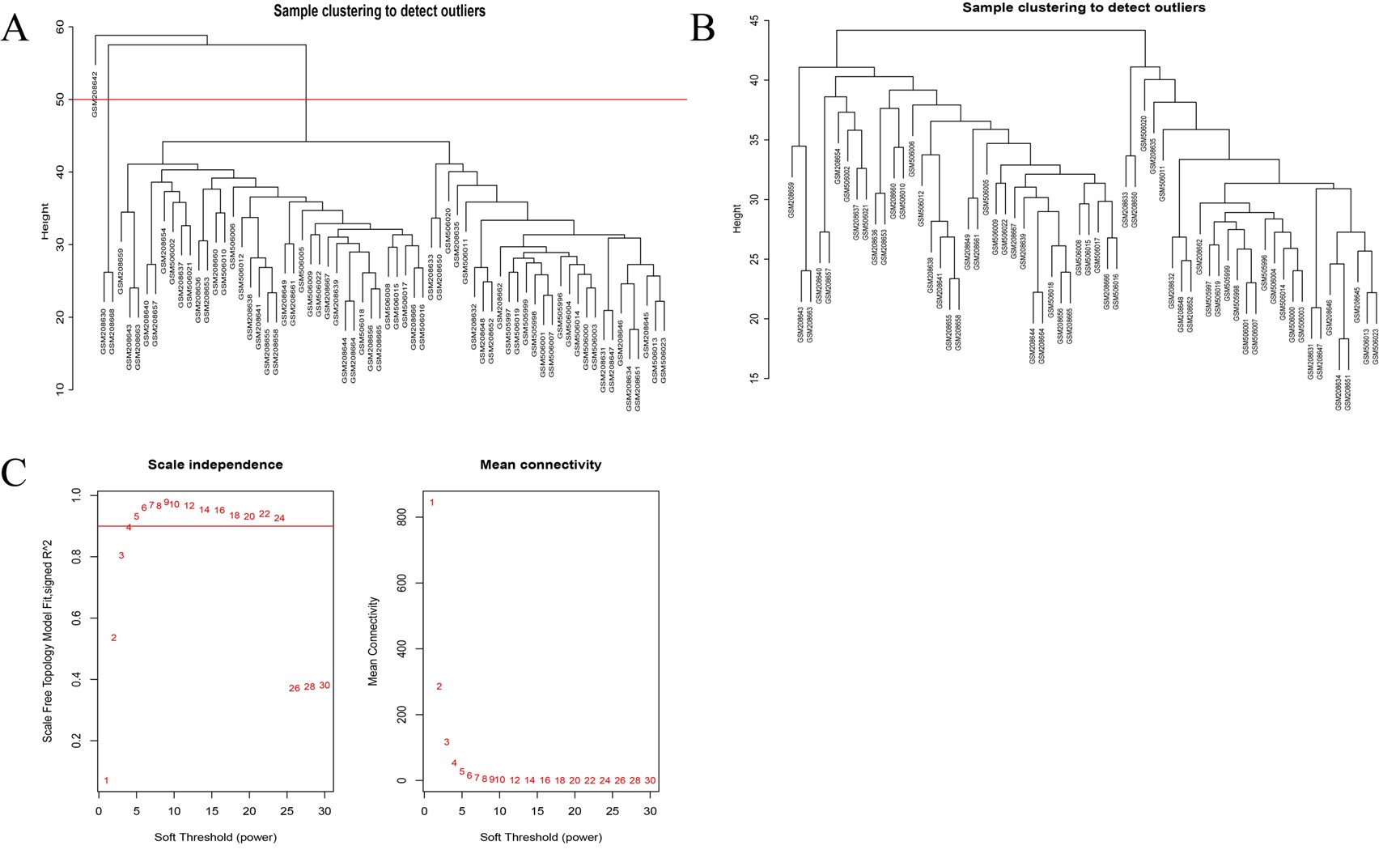

Supplement: Supplementary file 1 [file Image_1.JPEG]

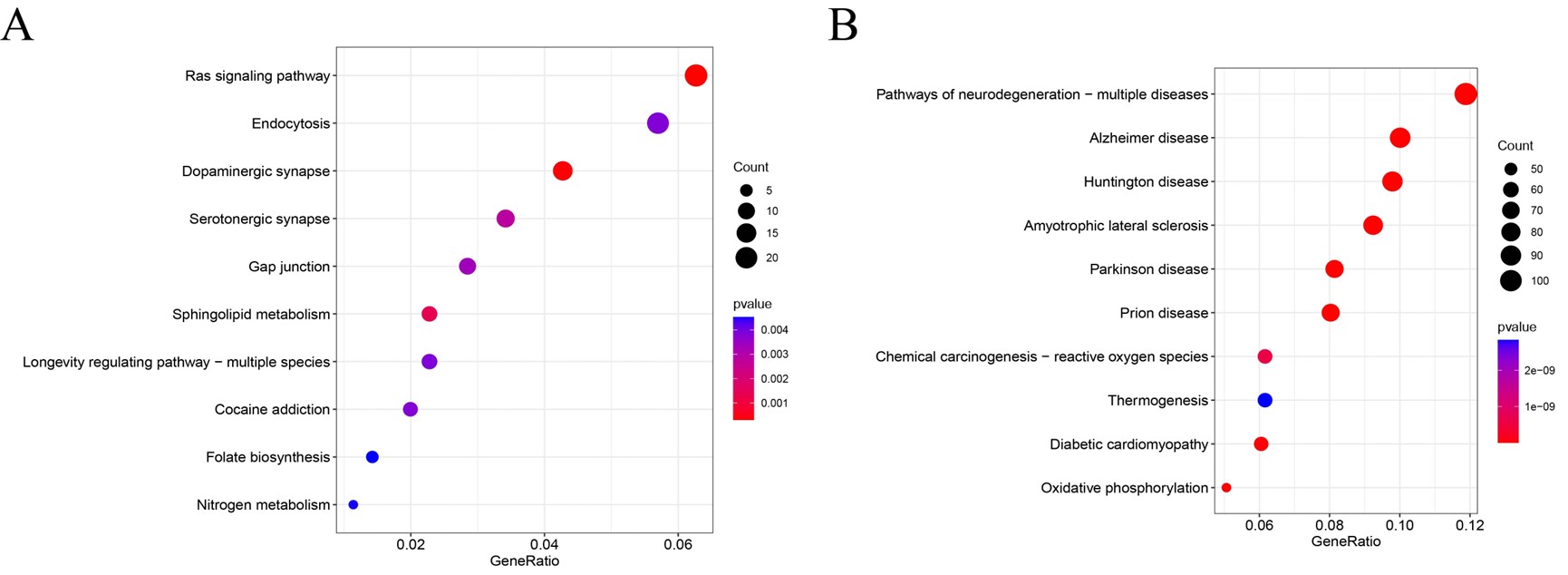

Supplement: Supplementary file 2 [file Image_2.JPEG]

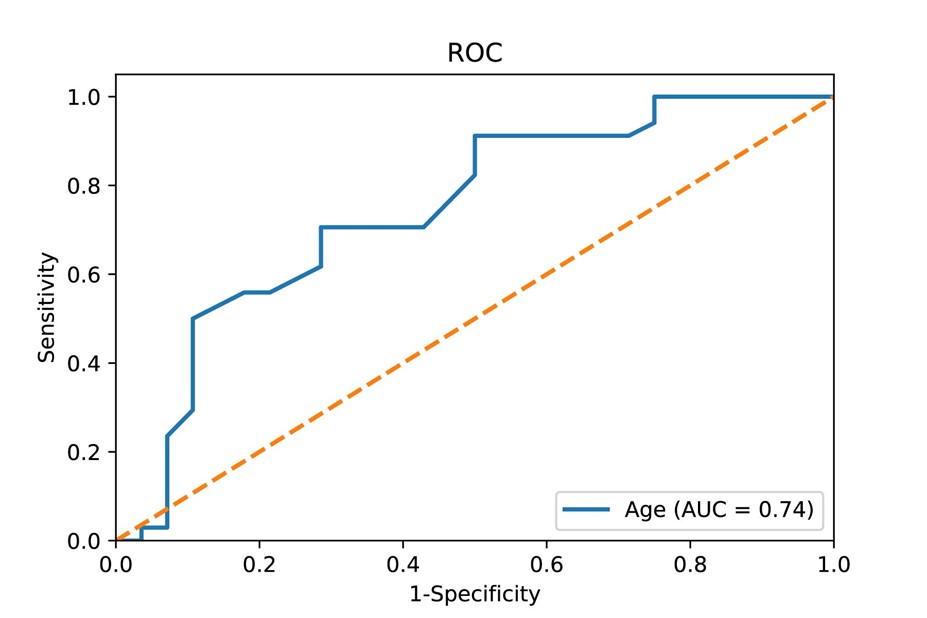

Supplement: Supplementary file 3 [file Image_3.JPEG]

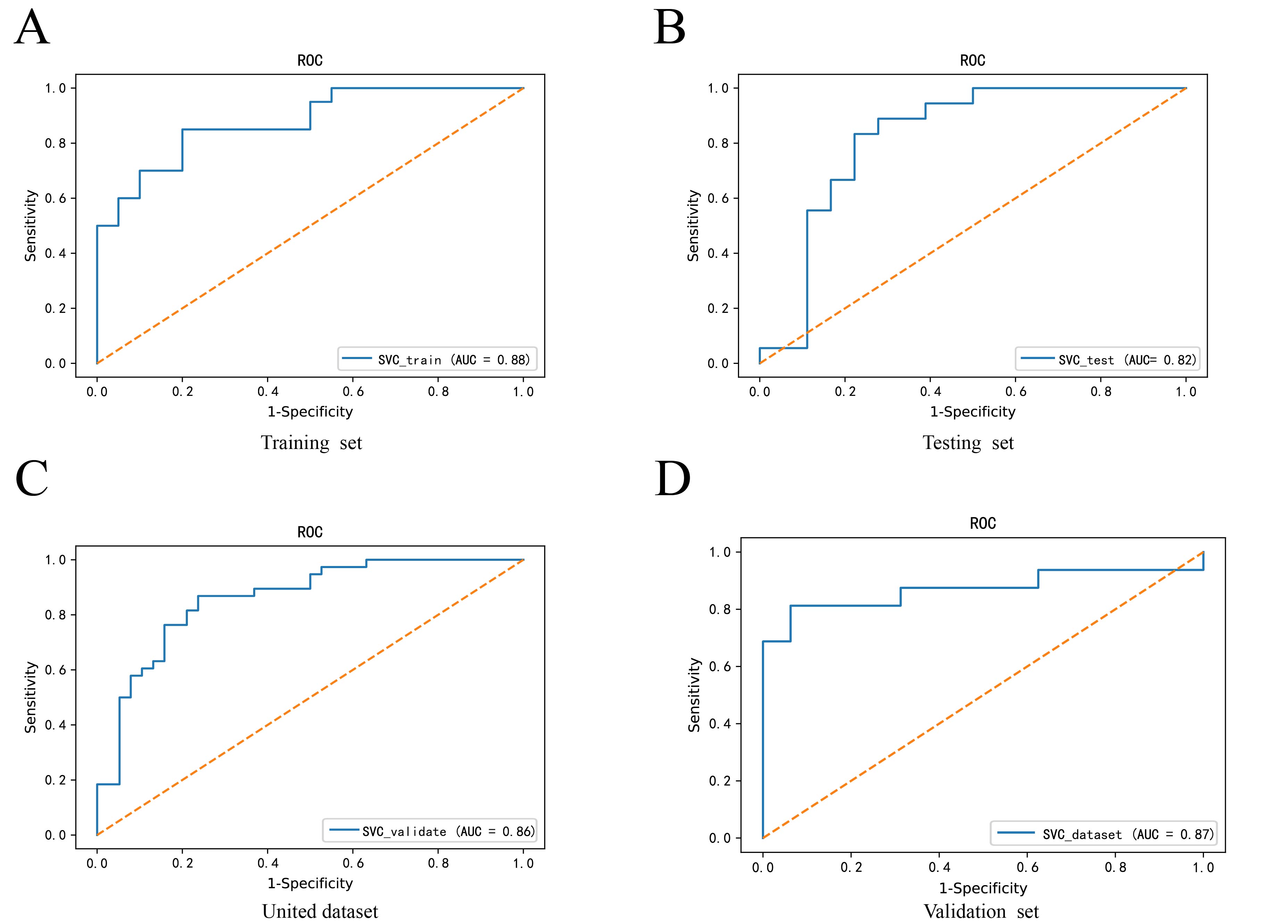

Supplement: Supplementary file 4 [file Image_4.JPEG]
