# Supplementary material for: The application of weighted gene co-expression network analysis and support vector machine learning in the screening of Parkinson’s disease biomarkers and construction of diagnostic models
Source: Front Mol Neurosci. 2023 Oct 16;16:1274268. doi: 10.3389/fnmol.2023.1274268 (PMC10614158; doi:10.3389/fnmol.2023.1274268)
Supplement: Supplementary file 5 [file Table_1.docx]

**Supplementary Table 1 Correlation coefficient between hub genes in the control stage**

|  | FLT1 | ATP6V0E1 | ATP6V0E2 | H2BC12 |
| --- | --- | --- | --- | --- |
| FLT1 | 1.00 | 0.55 | -0.26 | 0.31 |
| ATP6V0E1 | 0.55 | 1.00 | -0.49 | 0.82 |
| ATP6V0E2 | -0.26 | -0.49 | 1.00 | -0.46 |
| H2BC12 | 0.31 | 0.82 | -0.46 | 1.00 |
